# Modifying Effect of Smoking on GSTM1 and NAT2 in Relation to the Risk of Bladder Cancer in Mongolian Population: A Case-Control Study

**DOI:** 10.31557/APJCP.2021.22.8.2479

**Published:** 2021-08

**Authors:** Shiirevnyamba Avirmed, Yerkhanat Khuanbai, Amarsaikhan Sanjaajamts, Baasansuren Selenge, Bayan-Undur Dagvadorj, Makoto Ohashi

**Affiliations:** 1 *Division of Urology, School of Medicine, Mongolian National University of Medical Sciences, Ulaanbaatar, Mongolia. *; 2 *Department of Graduate Studies, Graduate School, Mongolian National University of Medical Sciences, Ulaanbaatar, Mongolia. *; 3 *Center of Urology and Andrology, First Central Hospital of Mongolia, Ulaanbaatar, Mongolia. *; 4 *Institute of Arts and Sciences, Tokushima University, Japan. *

**Keywords:** Bladder cancer, glutathione S-transferase M1, N-acetyltransferase 2, Smoking, Mongolia

## Abstract

**Objectives:**

Tobacco smoking is the predominant risk factor for bladder cancer as it contains cancer-causing chemicals. However, genetic factors may play important role in response towards chemical carcinogens. In this study we aim to investigate genetic polymorphisms of glutathione S-transferase M1 (GSTM1) and N-acetyltransferase 2 (NAT2) as determinants of bladder cancer risk, independently and in combination with tobacco use in the Mongolian population.

**Materials and Methods:**

The current study was a hospital-based case-control study including 60 histologically confirmed bladder cancer patients and 60 cancer-free controls. PCR-RFLP assay was used to determine the presence of GSTM1 and NAT2 polymorphisms in bladder cancer patients and controls. GSTM1 and NAT2 were tested using binary logistical regression analysis with adjustment or stratification according to the smoking.

**Results:**

There were 46 men and 14 women diagnosed with bladder cancer, with mean age was 58±4. The controls included 37 men and 23 women with a mean age of 57±3. The frequency of GSTM1 null genotype was higher in controls (71.67%) than in bladder cancer patients (58.33%) without statistical significance (OR=0.5534; 95% CI=0.2586-1.1843), (p=0.128). The NAT2 low acetylator phenotype was more common in patients with bladder cancer (15%) than in controls (5%). Furthermore, individuals with NAT2 low acetylator phenotype had a nearly 3.35-fold increased risk to develop bladder cancer (OR=3.35; 95% CI=0.8604-13.0657), (p=0.081) while the risk was even higher when combined with null GSTM1 genotype (OR=4; 95% CI=0.4459-37.5308), (p=0.213) but there was no statistical significance. Prevalence of smoking in bladder cancer patients was higher than controls and increased significantly the risk of bladder cancer (OR=8.31; 95% CI=3.66-18.88). Smokers with GSTM1 null genotype were at 5-fold higher risk of bladder cancer (OR=5.0; 95% CI=1.55-16.16), (p=0.007) while NAT2 low acetylator phenotype increased bladder cancer risk by 20-fold (OR=20.5; 95% CI=2.33-80.86), (p=0.006).

**Conclusion:**

The current study shows that tobacco smokers with the NAT2 low acetylator phenotype and GSTM1 null genotype have the highest risk of bladder cancer in the Mongolian population.

## Introduction

Bladder cancer is the tenth most common cancer in the world. It is the sixth most common cancer in males as well as the seventeenth in females, with approximately 549,000 new cases and 200,000 deaths each year (Bray et al., 2018). In Mongolia, bladder cancer is the second most frequent urological cancer and its incidence continues to increase (Sandagdorj et al. 2010). Tobacco smoking, occupational exposures, and environmental carcinogens are the most important risk factors for bladder cancer (Cumberbatch et al. 2015). Tobacco smoking is the predominant risk factor for bladder malignancy as roughly fifty percent of male urological cancer and thirty-three percent of female urological cancer can be attributed to tobacco smoking (Zeegers et al., 2000). Exposure to aromatic amines and polycyclic aromatic hydrocarbons (PAHs) have also been found to be related to bladder cancer susceptibility (Kogevinas et al., 2003). Aromatic amines (such as 4-aminophenyl) and PAHs (benzo[a]pyrene) were among sixty other carcinogens described in tobacco smoke (Hecht, 2003). However, while numerous individuals are exposed to these environmental factors, only a small percentage of exposed people develop cancer of the bladder. This suggests that genetic susceptibility may play a crucial role in bladder carcinogenesis when in combination with tobacco smoking and environmental exposures. 

Enzymes involved in the detoxification and metabolism of these carcinogens, such as glutathione S-transferases M1 (GSTM1) and N-acetyltransferases 2 (NAT2), are polymorphic in humans (Hung et al., 2004). Glutathione S-transferases are phase 2 detoxifying enzymes that play a role in the detoxification metabolism of environmental carcinogens by catalyzing their conjugation to glutathione and have been linked to the development of bladder cancer (Bell et al., 1993; Hayes and Pulford, 1995). The detoxification of polycyclic aromatic hydrocarbons and benzo(a)pyrene present in occupational exposures and tobacco smoke is aided by glutathione S-transferase M1 (Engel et al., 2002; Kim et al., 2002). The GSTM1 gene is located on chromosome 1p13.3, and homozygous deletion of this gene (GSTM1 null genotype) is the most common polymorphic variant characterized by a complete loss of enzyme activity (Lin et al., 1994). The distribution of GSTM1 null genotype is 38-67% in Europeans, 33-63% in Asians, and 22-35% in Africans and African-Americans (Rebbeck, 1997; Garte et al., 2001). Previous studies suggested that the GSTM1 null polymorphism has been linked with an increased risk of bladder cancer, especially among smokers (Engel et al., 2002; Garcia-Closas et al., 2005; Johns and Houlston, 2000; Salagovic et al., 1999). 

On the other hand, N-acetyltransferases encoded by 2 genes, NAT1 and NAT2, located on chromosome 8p22.These enzymes are expressed in the liver and involved in the detoxification of aromatic amines such as 4-aminobiphenyl and benzidine via O- and N- acetylation (Hein et al., 2000; Hein, 2009). The WT fast allele N-acetyltransferases2*4 and slow alleles N-acetyltransferases2*5A, 5B, 5C, 6, and 7 are the most common N-acetyltransferases 2 alleles (Meyer and Zanger, 1997). According to the intensity of acetylation, individuals with 2 WT alleles are classified as high acetylators, and those with 2 mutant alleles, such as M1, M2, or M3, are classified as slow-acetylators (Garcia-Closas et al., 2005). 

The distribution of slow acetylator phenotype is 5%, 50-60%, 90% among Canadian Eskimos, Caucasians and Northern Africans, respectively (Evans, 1989; Weber and Hein, 1985). Many epidemiological studies have indicated that NAT2 slow acetylation genotypes are linked with increased risk of bladder cancer, but the results are inconclusive (Golka et al., 2014). In current study, we examined whether polymorphisms of GSTM1 and NAT2 changed or accounted for the increased risk of bladder cancer independently and in combination with tobacco use among the Mongolian population.

## Materials and Methods

*Subjects*

Our study was a hospital-based case-control study including a group of sixty patients with histologically confirmed bladder cancer (46 men and 14 women, mean age 58±4) from the First Central Hospital of Mongolia and a group of sixty healthy controls (37 men and 23 women, mean age 57±3). The bladder cancer cases showed four histological forms including fifty-six transitional cell carcinoma, two squamous cell carcinoma, one adenocarcinoma and one bladder leiomyosarcoma. 

The control group was cancer-free healthy subjects with no family or personal history of bladder cancer. After obtaining written informed consent from a total of one hundred twenty subjects, all participants of the study were asked to fill out a questionnaire containing demographics, body mass index and tobacco use. In addition, 3 ml peripheral blood was collected from all subjects. This study was approved by the Ethics Committee of the Mongolian National University of Medical Sciences (№2019/3-13).

*DNA preparation and genotyping*

The Qiagen mini blood DNA extraction kit was used to extract genomic DNA from white blood cells (Qiagen, USA). Electrophoresis on a 1 percent agarose gel stained with EtBr was used to monitor the genomic DNA quality. An analysis of null GSTM1 gene polymorphisms was identified by multiplex PCR (Arand et al., 1996) and the N-acetyltransferases 2 gene polymorphisms were analyzed by PCR-RFLP technique. In brief, a Polymerase chain reaction amplification was done by digested fragment with restriction enzymes (KpnI, TaqI, BamHI) to detect slow acetylator alleles NAT2*5, NAT2*6 or NAT2*7. A single Polymerase chain reaction was conducted with the following primers: 5′-GGAACAAATTGGACTTGG-3′ and 5′-TCTAGCAT GAATCACTCTGC) (Bell et al., 1993). In an 80 L volume, 200ng of genomic DNA was applied to a PCR mix containing primers (50pM), dNTP (0.2 mM), Taq polymerase (1U), buffer of PCR, and MgCl2 (2.0 mM). It was then denatured for 240 seconds at 94°C before going through thirty cycles of half a minute at of 94°C, half a minute at 57°C, and 1 minute 30 seconds at 72°C. A last 300 seconds extension at 72°C was done, and the unique amplified fragment had a length of 1093 bp. Following Polymerase chain reaction, 0.02ml aliquots of the solution were digested with KpnI (for 60 minutes at 37°C; NAT2*5 allele), TaqI (for 60 minutes at 65°C; NAT2*6 allele) and BamHI (for 60 minutes at 30°C; NAT2*7 allele). The digested products were analyzed on agarose gels (2.0% (NAT2*5, NAT2*7) and 3.0% (NAT2*6) allele) and visualized by ultra-violet transilluminator. Subjects with 2 WT alleles were classified as high acetylators, and those with 2 mutant alleles, such as M1, M2, or M3, were classified as slow-acetylators (Vatsis et al., 1995). 

*Statistical analysis*

Differences of genotype and phenotype frequency in case and control groups were assessed by χ2 tests. Logistic regression was used to evaluate the risk of disease development and the results were adjusted by age and sex. The statistical analysis was performed by Stata 13.0 statistical software (Stata Corp, USA) and statistical significance was considered as a p value of ≤0.05.

## Results

*Subjects*

The baseline characteristics of bladder cancer patients and healthy controls are presented in Table 1. There were 46 men and 14 women diagnosed with bladder cancer, with a mean age was 58±4. The controls included 37 men and 23 women with a mean age of 57±3. In our study, the male to female bladder cancer prevalence ratio was 3.28:1. We found no statistical significance regarding age (p=0.575) and sex (p=0.075) between the bladder cancer patients and control group. The tobacco smoking prevalence among bladder cancer patients was significantly higher (71.67%) than in the control group (23.33%), (p<0.05). After adjustment for age and sex we found that tobacco smoking was associated with a significantly increased risk of bladder cancer, 8.3-fold (OR=8.31; 95% CI, 3.66-18.88) (p<0.000). 

*GSTM1 genotype and NAT2 phenotypes and bladder cancer risk*

The frequency of GSTM1 null genotype was higher in controls (71.67%) than in bladder cancer patients (58.33%), but without statistical significance (OR=0.55; 95% CI=0.26-1.18), (p=0.128). The NAT2 low acetylator phenotype was more common in patients with bladder cancer (15%) than in control group (5%). Furthermore, the individuals with NAT2 low acetylator phenotype had an almost 3.35-fold increased the risk of developing bladder cancer compared to individuals with NAT2 high acetylator phenotype (OR=3.35; 95% CI=0.86-13.07), (p=0.081). This risk was even higher when combined with GSTM1 null genotype (OR=4; 95% CI=0.45-37.58) although without statistical significance (p=0.213) (Table 2).

We evaluated the effect of GSTM1 genotype and NAT2 phenotypes on the risk of bladder cancer after adjustment by sex and age. The frequency of GSTM1 null genotype in females was slightly higher (73.09%) in control group than bladder cancer group, but there was no statistical significance (OR=0.27; 95% CI=0.07-1.09), (p=0.065). The distribution of GSTM1 null genotype in ≤60 years old individuals was higher in control group (OR=0.43; 95% CI=0.17-1.15), (p=0.094) while distribution of GSTM1 null genotype in ≥60 years old subjects was similar (OR=0.75; 95% CI=0.22-2.57) in both groups, but without statistical significance (p=0.647) (Table 3).

The NAT2 low acetylator phenotype distribution in males and females of the bladder cancer patients compared to control group was not significantly different (p=0.170) and (p=0.309) respectively. However, the NAT2 low acetylator phenotype increased the risk of developing bladder cancer (OR=3.14; 95% CI=0.61-16.13) and (OR=3.67; 95% CI=0.30-44.73) in both sexes respectively. The subjects at age ≤60 years old with NAT2 low acetylator phenotype were at an almost ten-fold increased the risk of developing bladder cancer than carriers of NAT2 high acetylator phenotype (OR=9.96; 95% CI=1.16-85.90) (p=0.037) (Table 4).

In bladder cancer patients the NAT2 high-acetylator phenotypes were WT/WT (46,67%), WT/M1 (25%), and WT/M3 (13.33%) where as M2/M2 (13.33%) was the most common low-acetylator phenotype (Table 5). The individuals with WT/M1 or M2/M2 phenotypes significantly increased 6.4-fold (OR=6.43; 95% CI=1.94-21.3) and 13.7-fold (OR=13.7; 95% CI=1.63-35.45) the risk of developing bladder cancer, respectively (p=0.002), (p=0.018).

Modifying effect of tobacco smoking on GSTM1 and NAT2 phenotypes in association with bladder cancer risk

Smokers with GSTM1 null genotype were at five-fold higher risk of developing bladder cancer (OR=5.0; 95% CI=1.55-16.16) while the risk was even higher with twenty-fold with NAT2 low acetylator phenotype (OR=20.5; 95% CI=2.33-80.86). The association between GSTM1, NAT2 phenotypes and the risk of bladder cancer was significant in smokers (p=0.007), (p=0.006) respectively (Table 6).

**Table 1:** Baseline Characteristics of Study Population

Variables	Controls (n=60)	Bladder cancer (n=60)	OR (95% CI)	p value
Age				
30-39	3 (5.01)	5 (8.33)		0.575
40-49	16 (26.6)	14 (23.3)		
50-59	17 (28.3)	11 (18.3)		
60-69	13 (21.6)	13 (21.6)		
70<	11 (18.3)	17 (28.3)		
Sex				
Male	37 (61.6)	46 (76.6)		0.075
Female	23 (38.3)	14 (23.3)		
BMI (kg/m^2^)				
18.5-24.9	23 (38.3)	14 (23.3)	1.00 (reference)	
25-29.9	15 (25.0)	19 (31.6)	2.10 (0.80-5.37)	0.13
30-39.9	21 (35.0)	24 (40.0)	1.97 (0.77-4.55)	0.163
40<	1 (1.63)	3 (5.03)	4.93 (0.46-52.1)	0.185
Tobacco use				
No	46 (76.7)	17 (28.3)	1.00 (reference)	
Yes	14 (23.3)	43 (71.7)	8.31 (3.65-18.9)	0.0001

**Table 2:** GSTM1 Genotype and NAT2 Phenotype in Relation Risk of Bladder Cancer

Variables	Controls (n=60)	Bladder cancer (n=60)	OR (95% CI)	p value
GSTM1				
Positive	17 (28.3)	25 (41.7)	1.00 (reference)	
Null	43 (71.7)	35 (58.3)	0.55 (0.26-1.18)	0.128
NAT2				
High	57 (95.0)	51 (85.0)	1.00 (reference)	
Low	3 (5.00)	9 (15.0)	3.35 (0.86-13.06)	0.081
GSTM1/NAT2				
GSTM1-pos/NAT2-high	15 (25.0)	22 (36.7)	1.00 (reference)	
GSTM1-pos/NAT2-low	2 (3.33)	3 (5.00)	1.02 (0.15-6.87)	0.982
GSTM1-null/NAT2-high	42 (70.0)	29 (48.3)	0.47 (0.21-1.06)	0.068
GSTM1-null/NAT2-low	1 (1.67)	6 (10.0)	4.09 (0.49-37.53)	0.213

**Table 3:** GSTM1 Genotypes among Bladder Cancer Patients and Controls after Adjustment by Sex and Age

Phenotype	Controls n (%)	Bladder cancer n (%)	OR (95% CI)	p value
Males				
Positive	11 (29.7)	17 (36.9)	1.00 (reference)	
Null	26 (70.3)	29 (63.1)	0.72 (0.28-1.81)	0.49
Females				
Positive	6 (26.1)	8 (57.1)	1.00 (reference)	
Null	17 (73.9)	6 (42.9)	0.26 (0.06- 1.08)	0.065
≥ 60 years				
Positive	11 (28.9)	16 (48.5)	1.00 (reference)	
Null	27 (71.1)	17 (51.5)	0.43 (0.16-1.15)	0.094
≥ 60 years				
Positive	6 (27.3)	9 (33.3)	1.00 (reference)	
Null	16 (72.7)	18 (66.7)	0.75 (0.21-2.57)	0.647

**Table 4:** NAT2 Phenotypes among Bladder Cancer Patients and Controls after Adjustment by Sex and Age

Phenotype	Controlsn (%)	Bladder cancer n (%)	OR (95% CI)	p value
Males				
High	35 (94.6)	39 (84.8)	1.00 (reference)	
Low	2 (5.41)	7 (15.22)	3.14 (0.61-16.13)	0.17
Females				
High	22 (95.7)	12 (85.7)	1.00 (reference)	
Low	1 (4.35)	2 (14.29)	3.66 (0.30-44.73)	0.309
≤ 60 years				
High	37 (97.4)	26 (78.8)	1.00 (reference)	
Low	1 (2.63)	7 (21.22)	9.96 (1.15-85.90)	0.037
≥ 60 years				
High	20 (90.9)	25 (92.6)	1.00 (reference)	
Low	2 (9.09)	2 (7.41)	0.82 (0.10-6.19)	0.831

**Table 5:** Distribution of NAT2 Genotypes among Bladder Cancer Patients and Controls

Genotype	Controls n=60	Bladder cancer n=60	OR (95% CI)	p value
WT/WT	48 (80.00)	28 (46.7)	1.00 (reference)	
WT/M1	4 (6.67)	15 (25.0)	6.42 (1.94-21.28)	0.002
WT/M2	1 (1.67)	0 (0)	1	
WT/M3	4 (6.67)	8 (13.3)	3.42 (0.96-12.42)	0.061
M1/M1	2 (3.33)	1 (1.67)	0.85 (0.07-9.88)	0.902
M1/M2	0 (0)	0 (0)		
M1/M3	0 (0)	0 (0)		
M2/M2	1 (1.67)	8 (13.3)	13.71 (1.62-35.45)	0.016
M2/M3	0 (0)	0 (0)		

**Table 6:** Modifying Effect of Smoking on GSTM1 and NAT2 in Relation to the Risk of Bladder Cancer

Variables	Controls n (%)	Bladder cancer n (%)	OR (95% CI)	p value
GSTM1/Smoking				
GSTM1-pos/non-smoker	15 (25.0)	6 (10.0)	1.00 (reference)	
GSTM1-pos/smoker	2 (3.33)	19 (31.7)	23.75 (4.17-34.98)	0
GSTM1-null/non-smoker	31 (51.7)	11 (18.3)	0.89 (0.27-2.85)	0.841
GSTM1-null/smoker	12 (20.0)	24 (40.0)	5 (1.54-16.16)	0.007
NAT2/Smoking				
NAT2-high/non-smoker	44 (73.3)	15 (25.0)	1.00 (reference)	
NAT2-high/smoker	13 (21.7)	36 (60.0)	8.12 (3.42-19.26)	0
NAT2-low/non-smoker	2 (3.33)	2 (3.33)	2.93 (0.37-22.68)	0.303
NAT2-low/smoker	1 (1.67)	7 (11.7)	20.53 (2.33-80.86)	0.006

## Discussion

Our current study investigates combined effect of tobacco smoking and GSTM1, NAT2 genotypes on the risk of developing bladder cancer in Mongolian population. Several studies have demonstrated the relationship between genetic polymorphisms of GSTM1, NAT2 and bladder cancer risk (Garcia-Closas et al., 2005). In our study, the GSTM1 null genotype was more common in healthy controls (71.67%) than in bladder cancer patients (58.33%) without statistical significance (OR=0.55; 95% CI=0.26-1.18), (p=0.128). This result was similar to that observed among healthy Asiatics (33-69%), and higher than that among the healthy population of Caucasians (55-60%), African-Americans (23-41%), Europeans (39-62%), Brazilians (48.86%) and Australians (51-54%) (Losi-Guembarovski et al., 2002). 

Furthermore, we identified no significant association between the GSTM1 null genotype and the risk of bladder cancer which is similar to the study by (McGrath et al., 2006). We have shown, however, that GSTM1 null genotype in combination with tobacco smoking (OR=5; 95% CI=1.55-16.16) increase significantly the risk of bladder cancer (p=0.007). It has been reported by several investigations (Engel et al., 2002; Garcia-Closas et al., 2005) that the GSTM1 null genotype increases the risk of bladder cancer, especially among smokers (Salagovic et al., 1999). 

The NAT2 slow acetylator phenotype was more common in patients with bladder cancer (15%) than in healthy controls (5%) in our study, which was similar to that observed among Northeast Asian countries (China, Japan, Taiwan, North Korea and South Korea) (18% on average) and lower than that among Europeans (59%), Caucasians, African-Americans (40- 70%) and Egyptians (80%) (Sabbagh et al., 2011). Moreover, in our study, smokers with NAT2 low acetylator phenotype increased twenty-fold the risk of developing bladder cancer. In Northeast Asian countries, the reason for low prevalence of NAT2 low acetylator phenotype is unknown but it has been speculated that differences in dietary habits or the chemical, physical environment, and high prevalence of the fast NAT2*4 (WT) haplotype may be contributing factors (Sabbagh et al., 2011). It has been hypothesized that tobacco smoke contains aromatic and heterocyclic carcinogens which are the important metabolites of NAT enzymes. Therefore, an increase in the amount of electrophilic-active products causes local somatic mutations in oncogenes, resulting in the start of tumor progression. Some studies showed that cytochromes P450 activated 2-amino-1-methyl-6-phenylimidazo [4,5-b] pyridine, the most abundant heterocyclic amine in cooked meat, beer and cigarette, further metabolized by NAT enzymes, which in turn results to the increased mutation frequency and tumor incidence (Bendaly et al., 2009). Interestingly, the subjects at age ≤ 60 years old with NAT2 low acetylator phenotype were at almost ten-fold increased risk to develop bladder cancer than carriers of NAT2 high acetylator phenotype (OR=9.96 95% CI=1.16-85.90, p=0.037) with statistical significance. 

In current study, the N-acetyltransferases 2 genotypes related with a high-acetylator phenotype commonly observed in bladder cancer patients were WT/WT (46,67%), WT/M1 (25%), WT/M3 (13.33%) and M2/M2 (13.33%) was the genotype most common related with the low-acetylator phenotype. Generally, the genetic polymorphisms of the NAT2 enzyme expression are the results of the four mutant alleles and NAT structural genes, and subsequently, the activity of this enzyme always depends on the combination of these alleles. It has been demonstrated by several studies that, presence of at least one WT can result in the high acetylator phenotype, while two mutant alleles might play high cancer susceptibility (Deguchi et al., 1990). Accordingly, our data suggests that genotype WT/M1 and M2/M2 may be a risk factor for cancer of the bladder in Mongolian population.

In conclusion, our current study shows that in the Mongolian population, the risk of developing bladder cancer was the highest for tobacco smokers with the NAT2 low acetylator phenotype and GSTM1 null genotype. Also, the risk of developing bladder cancer was higher for the subjects at age ≤ 60 years old with NAT2 low acetylator phenotype. As for NAT2 genotypes the individuals with WT/M1 and M2/M2 genotypes were at a 6.4-fold and 13.7-fold increased risk of developing bladder cancer, respectively.

## Author Contribution Statement

SA, MO and YK conceived the project and designed the research. SA, MO, YK, BS, AS and BD contributed to study conception, planning experiments and technical support. SA and YK conducted data analysis and interpretation, and wrote the manuscript. All authors read and approved the final manuscript. 

## Data Availability

All materials are available by the corresponding author.
